# IPS Interest in the EEG of Patients after a Single Epileptic Seizure

**DOI:** 10.1155/2016/5050278

**Published:** 2016-08-21

**Authors:** Fatima Zahra Taoufiqi, Jamal Mounach, Amal Satte, Hamid Ouhabi, Aboubaker El Hessni

**Affiliations:** ^1^Unit of Nervous and Endocrine Physiology, Laboratory of Genetics and Neuroendocrine Physiology, Department of Biology, Faculty of Sciences, Ibn Tofail University, PB 133, 14000 Kenitra, Morocco; ^2^Neurophysiology Department, Mohamed V Teaching Military Hospital, Rabat 10100, Morocco; ^3^Service of Neurology, Cheikh Khalifa Hospital, Casablanca 82403, Morocco

## Abstract

*Objective*. This study aims to evaluate the incidence of pathological cerebral activity responses to intermittent rhythmic photic stimulation (IPS) after a single epileptic seizure.* Patients and Methods.* One hundred and thirty-seven EEGs were performed at the Neurophysiology Department of Mohamed V Teaching Military Hospital in Rabat. Clinical and EEG data was collected.* Results.* 9.5% of our patients had photoparoxysmal discharges (PPD). Incidence was higher in males than in females, but *p* value was not significant (*p* = 0.34), and it was higher in children compared to adults with significant *p* value (*p* = 0.08). The most epileptogenic frequencies were within the range 15–20 Hz. 63 patients had an EEG after 72 hours; among them 11 were photosensitive (*p* = 0.001). The frequency of the PPR was significantly higher in patients with generalized abnormalities than in focal abnormalities (*p* = 0.001). EEG confirmed a genetic generalized epilepsy in 8 cases among 13 photosensitive patients.* Conclusion*. PPR is age related. The frequencies within the range 15–20 Hz should inevitably be included in EEG protocols. The presence of PPR after a first seizure is probably more in favor of generalized seizure rather than the other type of seizure. PPR seems independent from the delay Seizure-EEG. Our study did not show an association between sex and photosensitivity.

## 1. Introduction

Photosensitivity (PS) is clinically defined as an abnormal sensitivity of the brain in response to intermittent photic stimulation (IPS) [1], which is called PPR. PS can be assessed with different diagnostic procedures, but the most common method is IPS. A widely used EEG classification system was proposed by waltz, subclassifying PPR in four phenotypically different types [2, 3]:

 PPR type I: spikes within the occipital background activity;
 PPR type II: parietooccipital spikes and biphasic slow waves;
 PPR type III: parietooccipital spikes and biphasic slow waves spreading to frontal regions;
 PPR type IV: generalized spikes or polyspikes and waves.

There are few studies on the interest of IPS in the EEG in patients after a single epileptic seizure unlike the many studies dealing with photoparoxysmal responses to photic stimulation in epileptic patients and normal subjects [4–9].

The aim of this study is to evaluate the incidence of photosensitivity after a single seizure, the nature of these responses, and ILS frequencies that cause PPR.

## 2. Methods

This prospective study is related to patients admitted within a 29-month period (from 30/05/2010 to 09/04/2013). One hundred thirty-seven EEGs were performed in the ward of Neurophysiology Department of Mohamed V Teaching Military Hospital in Rabat. The patients' age ranged from 6 months to 84 years, with 64 being under age of 18 years. The study involved 59 females and 78 males. All of the patients had the EEG after a first seizure. Patients coming for consultation to this hospital were predominantly male soldiers.

A clipboard including the following information has been elaborated for each patient separately:

- sociodemographic data: age, sex, marital status, place of residence (home, nursing home, or another institution), and living alone or with others (children or other family members);
- past medical history, familial history of epilepsy, personal history of febrile convulsions, known risk factors for epilepsy seizure, intake of antiepileptic drugs (AEDS), a history of head trauma, metabolic disorder, vascular disease, psychiatric, and other diseases;
- clinical examination findings (EEG, brain imaging: CT-scan and/or MRI findings) were classified as normal or abnormal. EEG results: the following information was obtained from the available EEG reports/traces: PPR type, % EEGs with a PPR, epileptiform discharges during HV, ictal findings, and the range of IPS frequencies inducing a PPR.

### 2.1. EEG Procedure

The equipment of EEG is classified under the type of Deltamed Coherence version (2009) and is made up of electrodes for detection, equally divided on the scalp according to the international 10–20 system. Bipolar montage was used. EEG lasted thirty minutes in a calm atmosphere to assess the patients' reactivity through the use of hyperventilation (HV) and photic stimulation tests.

### 2.2. IPS Procedure

The procedure was performed in a dimly lit environment (but the observation window admitted enough light for viewing the subject), at least 3 min after hyperventilation. Photic stimulation performed SN 22 BB 183 (Braintonics BV, Gildemark 130, 1351 HL Almere, The Netherlands, Flash 201) photic stimulator. Recommended distance between the stroboscopic light and the patients nasion is 30 cm. Ten-second trains of flashes for each frequency were delivered, at intervals of ≥7 s. Eyes were kept open for the first 5 s and fixed at the center of the lamp. The patient was then asked to close the eyes and remain in the eyes-closed condition for the remaining 5 s of the stimulation. Recommended frequencies and their order of delivery are 2, 5, 10, 15, 20, 25, 30, 35, 40, 45, and 50 Hz (1 Hz = 1 flash/s). The total duration was a maximum of 6 min (patients without reaction to IPS). If an epileptiform discharges occurred, the stimulator was switched off, and the procedure was stopped.

### 2.3. Statistic Analysis

Epi info 3.5.1 was performed. A *p* value less than 10% was considered as significant.

## 3. Results

137 patients (59 females and 78 males; 73 adults and 64 children) were addressed for their first epileptic seizure. EEG was abnormal in 61 cases. IPS was ineffective in 124 subjects (90.5%). EEG allowed us to detect a photosensitivity with a photoparoxysmal response in 13 (9.5%) cases. Patients' ages ranged between 3.5 and 57 years. EEG performed after 72 h showed PPR in 11 cases; however, EEG obtained within 72 h did not show PPR (*p* = 0.001).

PPR was found in 11.5% males and 6.8% females (5.47% adults and 14.06% children) (Tables 1 and 2 present the details of the patients' data) (Tables 3, 4, 5, and 6 show the family and personal history of patients, photosensitivity, and other examinations performed).

The evolutionary timing between first seizure and EEG recordings for these 13 patients extended from 7 days to 3 years with an average of one year and a median of 150 days, of which the evolutionary timing of two cases could not be explained.

The EEG of these 13 (100%) patients confirmed generalized abnormalities in 10 cases (76.9%) and focal abnormalities in 2 cases (15.4%). One case (7.7%) presented in the first EEG few bihemispheric abnormalities during ILS and HV but a second sensitized EEG extended after 15 days was normal.

**Figure 1:**
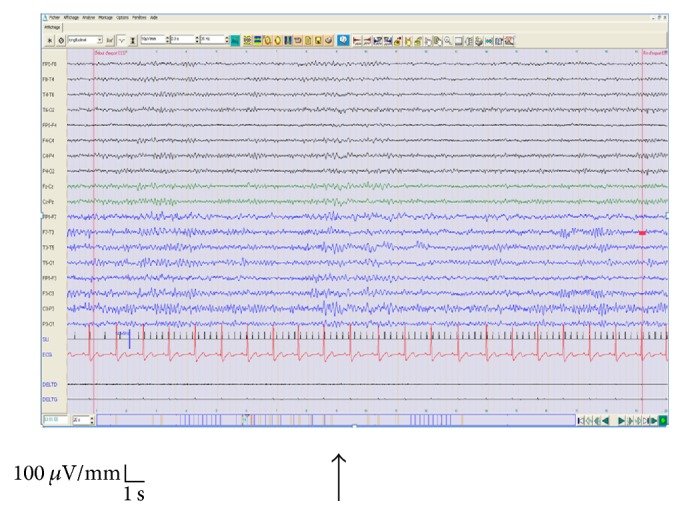
A fifty-seven-year-old female. Keppra (Levetiracetam) medication. Clinical data: loss of consciousness. Antecedent: operated for the month January 2012, left convexity meningioma. Operated for the month August 2011, intracranial brain frontotemporal left empyema. Routine EEG: normal background activity, abnormal spike-wave in left frontocentral which are broadcast sometimes in the right hemisphere activated by ILS (5 Hz) and by hyperventilation.

Of these 13 photosensitive cases (100%), 7 cases (53.8%) suffered from abnormalities by IPS and HV (4 cases were with genetic generalized epilepsy, 1 case presents a status epilepticus, 1 case has epileptic left frontotemporal abnormalities (Figure 1), and 1 case presented a bifrontal interictal epileptic abnormalities predominant in the left hemisphere) and 4 cases (30.8%) had abnormalities activated only by IPS (1 case was with Doose syndrome “myoclonic-astatic seizures” (Figure 3), 2 cases had a genetic generalized epilepsy, and 1 case presents a juvenile myoclonic epilepsy “JME”; this same case (underage girl) presented eyelid myoclonus and limbs sometimes accompanied by polyspikes waves on EEG).

**Figure 2:**
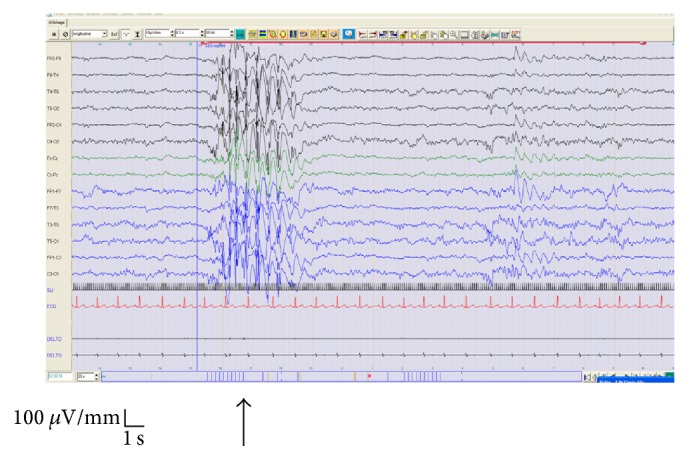
Eleven-year-old female. Clinical data: grand mal seizure on awakening, second degree of consanguinity. Antecedent: febrile syndrome at the age of 4 months. Routine EEG: generalized polyspikes and waves during ILS (15 Hz) and activated by hyperventilation. Diagnosis: genetic generalized epilepsy.

EEG of two other cases, which had no abnormality at the beginning of the graphics, showed abnormalities during IPS, which permitted the diagnosis (1 case (7.7%) had a genetic generalized epilepsy, epilepsy with GTCS on awakening (Figure 2), and 1 case (7.7%) had abnormalities during IPS with rare bihemispheric abnormalities).

**Figure 3:**
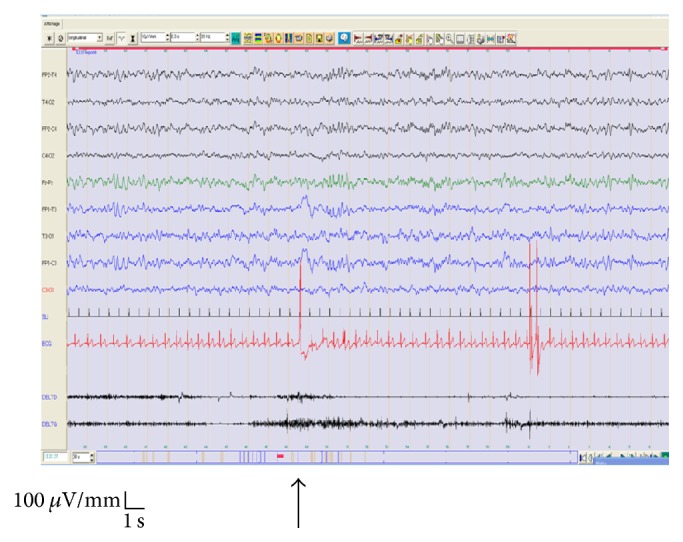
Six-year-old male. Depakine (valproic acid) medication. Clinical data: atonic seizure with sudden drop head trauma. He is an adopted boy since the age of 7 months (parents unknown). Routine EEG: registration of spike-wave speed to 4 c/sec diffuse activated by ILS at low frequency 2 Hz. Diagnosis: Doose syndrome.

The majority of the patients (11/13; 84.63%) expressed a PPR type IV (Figures 2 and 3) during IPS and 2/13 (15.4%) expressed a PPR type III (Figure 1). PPR types I and II were not found in any of our cases. Most patients [6  (46%)] were sensitive at an IPS range of 15–20 Hz, 5 (38%) patients were sensitive at 2 Hz, and 2 (16%) patients were sensitive at 5 Hz, respectively.

## 4. Discussion

 Berger first described a change in EEG activity following a light stimulus [10]. This finding was confirmed by Adrian and Matthews [11] who showed that a flickering light sometimes resulted in rhythmical oscillation of the occipital brain waves [12]. There is almost general agreement that the EEG abnormalities induced by IPS in photosensitive patients are generalized or starting in the posterior cerebral regions, especially the occipital regions [13, 14]. It has been suggested that a single epileptic seizure in a patient is associated with increased cerebral neuronal excitability as reflected by the PPD [14]. Our data showed that in photosensitive patients, PPD responses evoked by IPS were generalized in 76.9% of cases (*p* = 0.001).

PPD in our study was more frequent comparing to other studies [15] (9.5% versus 6.4%).

Among the patients with single seizures, PPD was higher in males compared to females with no significant *p* value (*p* = 0.34) and in children compared to adults with significant *p* value (*p* = 0.08). A number of studies have reported the higher incidence of PPD in females compared to males [15–18]. This observation was thus confirmed not only in patients with single seizures but also in epileptic patients [15] and the peak age was during adolescence [19]. In our study, males outnumbered females. This is because of recruitment bias: the consulting population is made mostly of male soldiers. Studies in epileptic patients show that an epileptiform response to IPS is found in about 10–20% of children and 5–10% of adults [3]. Another study reported a higher incidence of pathological responses in the younger population [20] and is most frequently seen in the first decades of life [18]. Another study suggests that individual factors such as age and gender but also other unknown factors influence the expression of the PPR [1, 21]. In fact It has been suggested that photoparoxysmal response is an age and a sex related phenomenon and this fact should be considered when IPS is evaluated; therefore, It was proposed that, without change in medication, photosensitivity disappears as the patients become older [22].

None of the 21 patients with MRI or CT-scan abnormalities had PPR induced by IPS, which suggests that photosensitivity is not related to brain lesions or damage. De Kovel et al. [23] showed in his mega-analysis that PPR had a strong genetic basis. However, in our study family and personal history of patients did not alter the response to IPS. No conclusion can be made as the number of patients is small. It has been pointed out that the presence of PPD in patients after single epileptic seizure is probably associated with increased cerebral neuronal excitability [22].

The evolutionary timing between first seizure and EEG recordings for our 13 patients extended from 7 days to 3 years with an average of one year. The evolutionary time for 2 cases could not be explained. The diagnosis timing can be difficult to quantify. The majority of our patients do not give a precise date of the inaugural seizure. In daily practice, answers such as “there are four months, there is almost a year…,” are common when patient or a family member is asked about the date of the first crisis. This delay in the consultation can also be explained by ignorance of the patients or their parents' relatives who firstly use traditional methods.

IPS was ineffective in 124 subjects (90.5%). 63 patients had an EEG after 72 h. Among them 11 were photosensitive. It is known that EEG within 24 h of the seizure is more useful to detect ictal and interictal abnormalities. Our study shows that PPR was frequent even after 72 hours (*p* = 0.001). PPR seems, thus, independent from the delay Seizure-EEG contrary to interictal abnormalities. King et al. [24] reported that EEG within 24 h was more useful in diagnosis of epileptiform abnormalities than later EEG (51% versus 34%) and concluded in their study that an EEG should be obtained within 24 h of the seizure followed by a sleep deprived EEG if necessary. Sleep deprivation appeared to be more effective as an activating method of EEG [25].

In our study, EEG of our 13 (100%) patients confirmed genetic generalized epilepsy in 8 (61.5%) cases, structural/metabolic or unknown generalized epilepsy in one case (7.7%) (Doose syndrome), and a generalized status epilepticus in 1 (7.7%) case. Lu et al. [18] reported that photosensitivity was more common in idiopathic generalized epilepsy (epilepsy with grand mal on awaking, 74%; juvenile absence epilepsy, 56%; juvenile myoclonic epilepsy, 50%; childhood absence epilepsy, 44%) than in focal types (idiopathic partial-rolandic epilepsy, 23%; symptomatic/cryptogenic type of epilepsy 16%). An epilepsy syndrome can be diagnosed in most first seizure patients [24]. Olafsson et al. [26], however, reported that patients presented with a first diagnosis of a single unprovoked seizure and epilepsy; genetic epilepsy syndromes were identified in 14% of all patients. Another study concluded that PPR is most commonly associated with IGEs such as juvenile myoclonic epilepsy (JME) [21].

In our study, the epileptogenic frequencies were within the range 15–20 flashes/s (46%); 38% of the photosensitive population is sensitive at 2 flashes/s. Zifkin and Inoue [27] reported that only 3% of the photosensitive population is sensitive at 1–3 flashes/s and the flash frequencies most likely to elicit a PPR to IPS range typically from nine to 18 flashes/s. Another study found that the most epileptogenic frequencies were within the range 15–18 Hz [28]. Takahashi and Tsukahara [29] noted that flicker frequency of 20–15 Hz was most effective in eliciting generalized PPR. Other surveys found that most patients are sensitive between 10 and 30 Hz [30] and peak sensitivity is between 16 and 20 flashes/s [16]. Fisher et al., 2005, reported that frequencies of 15–25 Hz are the most provocative. The difference between our findings and those reported by others may be attributable to certain aspects of our technique: the combination of IPS and hyperventilation. Srinivasulu Naidu et al. [31] reported that despite being used in a routine clinical EEGs for decades, a number of different views on the usefulness and indications for these procedures exist. From clinical studies it is clear that variation of the frequency of the stimulus, as well as of other parameters, affects the response.

- The majority of the patients (11/13; 84,63%) expressed a PPR type IV during IPS and 2/13 (15,4%) expressed a PPR type III. Demirkaya et al. [3] reported that 84% of patients expressed a PPR type IV.

## 5. Conclusion

Our study suggests that photosensitivity is age dependent and is not linked to sex. No association between familial, personal history and photosensitivity was found.

The majority of our patients expressed types IV and III PPR and the most epileptogenic frequencies were within the range 15–20 Hz. These frequencies should inevitably be included in EEG protocols.

PPR seems to be independent from the delay Seizure-EEG contrary to interictal abnormalities.

## Figures and Tables

**Table 1:** Incidence of photoparoxysmal response to photic stimulation: patients with isolated seizures. Adults: those who had EEG records at the age of 18 years and above [19 years to 84 years]. Children: those who had EEG records before the age of 18 years [6 months to 17 years].

	Number	Number with PPD^*∗*^	%
*All patients*	137	13	9.5
*Males*	78	9	11.5
*Females*	59	4	6.8
*Adults*	73	4	5.47
*Children*	64	9	14.06
*Patients with:*			
*Generalized seizure:*			
Tonic-clonic	63	10	15.9
Tonic	6	—	—
Absence	7	—	—
Atonic	3	—	—
Myoclonic	2	—	—
Clonic	1	—	—
*Focal seizure (simple):*			
Occipital	1	—	—
Temporal	1	—	—
Frontal	2	—	—
*Secondarily generalized seizure*	2	—	—
*Indetermined semiology*	44	3	6.8
*Spasms*	1	—	—

^*∗*^Photoparoxysmal discharges, PPD in children > adults, PPD in males > females, and PPR IV > PPR III.

**Table 2:** Clinical data of patients.

Clinical data	Number	Abnormality on IPS
Normal	97	9
Confusion	2	0
Hemiparesis/hemiplegia	10	2
Headaches	13	0
Psychomotor delay	6	0
Behavior disorders	1	1
Mental retardation	2	0
Insomnia	1	0
Daytime sleepiness	1	0
Memory disorder	2	1
Posttraumatic encephalopathy + memory disorder	1	0
Frontal contusion	1	0

*Total*	137	13

**Table 3:** Family history of patients.

History	No abnormality on IPS	Abnormality on IPS	Total
Family history of epilepsy	16	0	16
Consanguinity	14	1	15
No family history	94	12	106

Total	124	13	137

**Table 4:** Personal history of patients.

Personal history	No abnormality on IPS	Abnormality on IPS	Total
Neonatal pain	1	0	1
Tumor brain	3	0	3
Head trauma	15	1	16
Cerebellar syndrome	1	0	1
Vascular + metabolic	5	0	5
Vascular	7	0	7
Inflammatory and metabolic history	1	0	1
Inflammatory history	1	0	1
Febrile convulsion	9	5	14
Head trauma + febrile convulsion	1	0	1
Febrile headache	1	0	1
Metabolic	7	0	7
Head trauma + metabolic	0	1	1
Sickle cell thalassemia	1	0	1
Tumor + intracranial empyema	0	1	1
Catatonic syndrome	1	0	1
Intracerebral cyst in left	1	0	1
Empyema + brain abscess	1	0	1
No personal history	68	5	73

Total	124	13	137

**Table 5:** EEG findings.

EEG findings	Number with PPR^*∗∗*^	%
Generalized abnormalities	10	76.9
Focal abnormalities	2	15.4
PPR type III	2	15.4
PPR type IV	11	84.6
IPS-sensitivity range (Hz) 2 (%)	5	38
IPS-sensitivity range (Hz) 5 (%)	2	16
IPS-sensitivity range (Hz) 15–20 (%)	6	46

^*∗∗*^Photoparoxysmal response; PPR IV > PPR III.

**Table 6:** MRI and CT scan examinations.

Additional tests	Number	Abnormality on IPS
*MRI:*		
(i) Cerebellar syndrome	1	—
(ii) Frontal left hemorrhagic contusion	1	—
(iii) Normal	4	—
(iv) Hyper T2 in right temporal lobe	1	—
(v) Left frontal contusion	1	—
(vi) Large hematoma	1	—
(vii) Arachnoid cyst of the left temporal lobe	1	—
(viii) Global cerebral atrophy	1	—

*CT-scan:*		
(i) Normal	6	—
(ii) Parietal lesions	1	—
(iii) Arteriovenous malformation in the right temporal lobe	1	—
(iv) Hypodense lesion	2	—
